# Supplementary material for: Changes in Default Mode Network Connectivity in Resting-State fMRI in People with Mild Dementia Receiving Cognitive Stimulation Therapy
Source: Brain Sci. 2021 Aug 27;11(9):1137. doi: 10.3390/brainsci11091137 (PMC8468883; doi:10.3390/brainsci11091137)
Supplement: Supplementary file 1 [file brainsci-11-01137-s001.zip › brainsci-1344075-supplementary.pdf]

Table S1. Correlations between age, cognitive reserve proxies, brain reserve proxy, and ADAS-Cog scores in CST and TAU groups

CST group (n=16)		2	3	4	5	6	7	8	9	10	11	12	13	14 T0T1 TG
1	Age, years	0	-0.45	-0.07	0.05	0.62*	0.26	0.33	-0.08	0.5	0.56*	0.47*	0.17	-0.42
2	Gender, female	--	-0.38	-0.28	0.56*	0.09	-0.04	0.19	0.08	0.05	0.34	0.03	0.43	0.31
3	Education, years		--	0.25	-0.47	-0.42	-0.37	-0.19	0.33	-0.21	-0.23	-0.15	-0.12	-0.03
4	Work, years			--	-0.02	-0.19	0.19	0.16	0.17	0.3	0.23	-0.27	0.45*	-0.33
5	Baseline TBV/TICV, %				--	0.09	0	0.38	-0.3	-0.21	0	-0.50*	0.57*	0.38
6	Baseline DMN rs-FC, *r*					--	0.45	0.16	0.18	0.2	0.2	0.31	-0.14	-0.02
7	Baseline CEN rs-FC, *r*						--	0.21	0.12	0.14	0.14	0.04	0.15	0.17
8	Baseline LAN rs-FC, *r*							--	-0.11	0.26	0.46	0.3	0.28	-0.11
9	Baseline CSDD								--	0.36	0.07	-0.03	0.14	-0.17
10	Baseline HCS									--	0.63*	0.47	0.24	-0.66**
11	Baseline ADAS-Cog total										--	0.72*	0.41	-0.34
12	Follow-up ADAS-Cog total											--	-0.34	-0.40
13	Change in ADAS-Cog total[a]												--	0.06
TAU group (n=13)		2	3	4	5	6	7	8	9	10	11	12	13	14 T0T1 TG
1	Age, years	-0.08	-0.48	0.1	0.02	0.02	-0.21	-0.1	0.22	0.58	0.29	0.47	-0.29	-0.31
2	Gender, female	--	0	-0.44	0.21	0.70**	0.4	-0.25	0.37	0.37	0.49	0.31	0.51	-0.11
3	Education, years		--	-0.43	-0.48	-0.22	-0.33	-0.25	-0.25	-0.05	-0.34	-0.13	-0.5	0.16
4	Work, years			--	-0.07	-0.03	-0.34	0.31	0.11	-0.2	-0.41	-0.42	-0.08	-0.33
5	Baseline TBV/TICV, %				--	0.04	0.51	0.3	0.03	-0.28	0.2	-0.08	0.63*	0.27
6	Baseline DMN rs-FC, *r*					--	0.35	-0.17	0.41	0.32	0.19	0.01	0.41	-0.29
7	Baseline CEN rs-FC, *r*						--	0.06	0.26	-0.03	0.47	0.3	0.68*	0.01
8	Baseline LAN rs-FC, *r*							--	-0.43	-0.46	-0.32	-0.41	0.12	0.16
9	Baseline CSDD								--	0.83*	0.35	0.31	0.18	-0.49
10	Baseline HCS									--	0.28	0.39	-0.45	-0.89**
11	Baseline ADAS-Cog total										--	0.90*	0.46	0.15
12	Follow-up ADAS-Cog total											--	0.03	0.01
13	Change in ADAS-Cog total[a]												--	0.31

CST = Cognitive Stimulation Therapy; TAU = treatment as usual; ADAS-Cog = Alzheimer's Disease Assessment Scale, Cognitive subscale; CSDD = Cornell Scale of Depression in Dementia; HCS = Holden Communication Scale; TICV = total intracranial volume; TBV = total brain volume; DMN = default mode network; CEN = central executive network; LAN = language network; rs-FC = resting-state functional connectivity; T0T1 TG = Time gap between baseline and follow-up behavioral assessments

*p<0.05; **p<0.01

[a]Positive values signify improvement on ADAS-Cog.

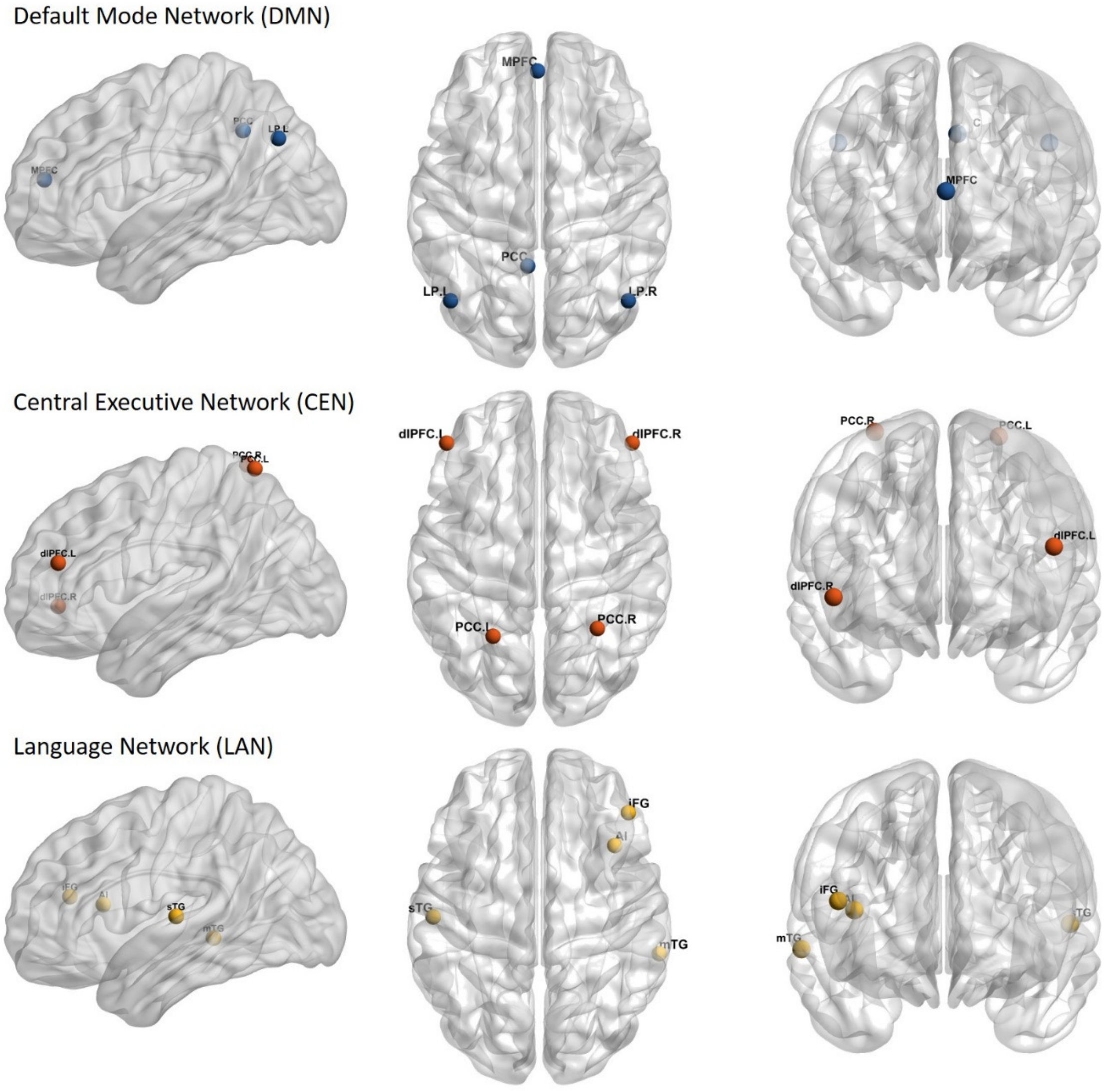

Figure S1. Regions of interest used in the functional connectivity analysis, superimposed on a three-dimensional brain template
